# Patulin Ameliorates Hypertrophied Lipid Accumulation and Lipopolysaccharide-Induced Inflammatory Response by Modulating Mitochondrial Respiration

**DOI:** 10.3390/antiox12091750

**Published:** 2023-09-11

**Authors:** Seulmin Hong, Seon Kyeong Park, Jangho Lee, Soo Hyun Park, Young-Soo Kim, Jae-Ho Park, Seungmin Yu, Yu Geon Lee

**Affiliations:** 1 Personalized Diet Research Group, Korea Food Research Institute (KFRI), Wanju 55365, Republic of Korea; h.seulmin@kfri.re.kr (S.H.); p.seonkyeong@kfri.re.kr (S.K.P.); jhlee@kfri.re.kr (J.L.); shpark0204@kfri.re.kr (S.H.P.); jaehopark@kfri.re.kr (J.-H.P.); 2 Department of Food Science & Technology, Jeonbuk National University, Jeonju 54896, Republic of Korea; ykim@jbnu.ac.kr

**Keywords:** patulin, obesity, inflammation, mitochondrial, oxidative function

## Abstract

Patulin (PAT) is a natural mycotoxin found in decaying pome fruits. Although some toxicological studies have been conducted on PAT, recent research has highlighted its anticancer and antifungal effects. However, studies have yet to examine the effects and molecular mechanisms of PAT in other metabolic diseases. Obesity is a chronic disease caused by excessive food intake and abnormal lifestyle, leading to low-grade inflammation. Therefore, this study aimed to elucidate the effect of PAT on obesity at the cellular level. PAT treatment reduced lipid accumulation, suppressed glucose and LDL uptake, inhibited lipid deposition and triglyceride synthesis, upregulated fatty acid oxidation-related genes (*Pgc1α*), and downregulated adipogenic/lipogenic genes (*Pparγ* and *C/ebpα*) in hypertrophied 3T3-L1 adipocytes. Additionally, PAT treatment enhanced mitochondrial respiration and mass in differentiated adipocytes and alleviated inflammatory response in activated RAW 264.7 macrophages. Moreover, PAT treatment downregulated pro-inflammatory genes (*il-6*, *Tnf-α*, *Cox-2*, and *inos*), suppressed lipopolysaccharide (LPS)-induced increase in inflammatory mediators (IL-6, TNF-α, and NO), and restored mitochondrial oxidative function in LPS-stimulated macrophages by improving oxygen consumption and mitochondrial integrity and suppressing ROS generation. Overall, these findings suggest a potential for PAT in the prevention of lipid accumulation and inflammation-related disorders.

## 1. Introduction

Obesity represents a significant health issue marked by excessive fat accumulation, arising from an imbalance between energy consumption, storage, and expenditure, and is intricately associated with the onset of several metabolic disorders, including insulin resistance, diabetes, and cardiovascular disease [1,2]. Additionally, obesity is mainly associated with increased white adipose tissue mass due to the activation of adipogenesis and elevated cytoplasmic triglyceride (TG) deposition [3]. Adipogenesis is a tightly regulated process, in which undifferentiated preadipocytes differentiate into mature adipocytes [4]. Adipogenesis is mainly governed by a complex network of transcription factors, including CCAAT/enhancer-binding protein (C/EBP) and peroxisome proliferator-activated receptor-γ (PPAR-γ), which stimulate fatty acid synthesis and transport to promote TG accumulation in mature adipocytes [5]. The activation of these transcription factors can upregulate the expression of key genes involved in lipid metabolism and adipocyte function, such as fatty acid synthase (FASN), fatty acid-binding protein 4 (FABP4), and ATP-citrate lyase (ACLY) [6]. Additionally, adipocytes secrete various adipokines, including adiponectin, which are involved in insulin signaling and glucose regulation [7]. The upregulation of adipogenesis and accumulation of mature adipocytes contribute to the disruption of fatty acid oxidation and glucose metabolism, subsequently compromising glucose homeostasis and insulin sensitivity [8]. Therefore, modulating processes that control adipocyte differentiation could be an effective strategy to prevent or treat metabolic diseases.

Obesity is considered a low-grade chronic inflammatory disease due to an imbalance between pro-inflammatory stimuli and anti-inflammatory immune response [9]. The release of pro-inflammatory molecules from adipose tissue is a significant mechanism that underlies the inflammatory nature of obesity [9]. Specifically, the infiltration of macrophages into adipose tissue is responsible for the secretion of several substances known as adipokines, including pro-inflammatory cytokines such as tumor necrosis factor-alpha (TNF-α) and interleukin-6 (IL-6) [10]. Additionally, infiltrating immune cells in adipose tissue, particularly macrophages, interact with adipocytes and further contribute to the development of chronic low-grade inflammation [11]. Moreover, adipose tissue produces anti-inflammatory molecules, including adiponectin, which exert beneficial effects on glucose metabolism and have the potential to reduce inflammation [12]. However, the ability of anti-inflammatory molecules to counteract inflammatory response is impaired in obesity [13]. Accordingly, a chronic inflammatory state has a detrimental impact on insulin resistance, dyslipidemia, hypertension, and other metabolic abnormalities associated with obesity [14]. Specifically, the crosstalk between macrophages and adipocytes is crucial for initiating and sustaining a chronic inflammatory state in obese adipose tissue [15]. Therefore, targeting their interaction could be an effective strategy for the treatment of obesity.

Mitochondria play a central role in energy metabolism by producing adenosine triphosphate (ATP) for the cells from food substrates, such as glucose and lipids [16]. In addition to ATP generation, mitochondria are involved in biosynthesis, including the production of fatty acids [17], and in the production and elimination of reactive oxygen species (ROS) [18]. In obesity, dysfunctional mitochondria can contribute to the development of metabolic abnormalities and obesity-related complications [17,18]. For instance, excessive food consumption leads to elevated levels of blood glucose, resulting in the overproduction of ROS within adipocyte mitochondria [19]. The increased mitochondrial ROS levels contribute to mitochondrial dysfunction, impairing their ability to perform essential metabolic processes, such as bioenergetics and biosynthesis pathways [20]. Consequently, obese adipose tissues are characterized by reduced mitochondrial mass and impaired mitochondrial oxidative capacity [21]. Additionally, an obesity-induced decrease in mitochondrial biogenesis further contributes to insulin resistance and low-grade inflammation [22]. Therefore, strategies that promote mitochondrial biogenesis and maintain mitochondrial quality could be effective in ameliorating the progression of obesity and associated metabolic disorders.

**Figure 1:**
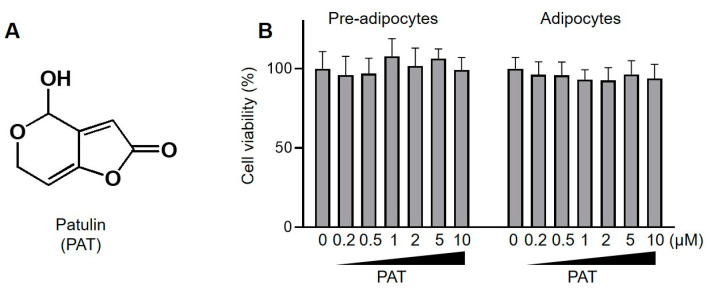
Effects of patulin (PAT) on the viability of 3T3-L1 adipocytes. (**A**) Chemical structure of PAT. (**B**) The 3T3-L1 preadipocytes were treated with different concentrations of PAT (0–10 μM, left bar graph). The 3T3-L1 preadipocytes were differentiated in a medium containing MDI in the presence of different concentrations of PAT for 8 days (right bar graph). Cell viability was measured using a WST-1 kit.

Patulin (PAT, Figure 1A) is a mycotoxin produced by various fungal species found in diverse food products, including fruits and vegetables [23]. Although toxicity studies have investigated the neurological side effects of PAT [24], some studies suggest that it possesses anticancer and anti-inflammatory effects [25,26]. Notably, PAT is a potent inducer of AMPK signaling in hepatocytes, suggesting that it may have a preventive effect on the development and progression of fatty liver diseases [27]. However, there is still a lack of information on the pharmacological effects of PAT on metabolic diseases, including obesity. Therefore, this study aimed to investigate the anti-inflammatory and antilipidemic properties of PAT at the cellular level.

## 2. Materials and Methods

### 2.1. Chemical and Reagents

PAT was obtained from MedChem Express (#HY-N6779, Monmouth Junction, NJ, USA). 3-(4,5-Dimethylthiazol-2-yl)-2,5-diphenyltetrazolium bromide (MTT) and other reagents were purchased from Sigma–Aldrich (St. Louis, MO, USA). Antibodies against PPAR gamma (16643-1-AP, Proteintech, Chicago, IL, USA), FASN (#sc-48357, Santa Cruz Biotechnology, Santa Cruz, CA, USA), ACLY (#15421-1-AP, Proteintech), PGC1 alpha (#PA1-31202, Invitrogen, Carlsbad, CA, USA), α/β-tubulin (#2148, Cell Signaling Technology, Danvers, MA, USA), and β-actin (#sc-47778, Santa Cruz Biotechnology, Santa Cruz, CA, USA) were used for Western blotting.

### 2.2. Cell Culture

The 3T3-L1 cells were purchased from the American Type Culture Collection (ATCC, Manassas, VA, USA). Cells were cultured in high-glucose Dulbecco’s Modified Eagle’s Medium (DMEM) (Welgene, Gyeongsan, Republic of Korea) containing 10% BCS (Welgene) and 1% penicillin-streptomycin (10,000 U/mL) solution (Gibco, Carlsbad, CA, USA) at 37 °C in a 5% CO_2_ atmosphere. To induce differentiation, confluent cells were incubated in DMEM containing 10% FBS (Welgene) and MDI (0.5 mM of IBMX, 1 µM of DEX, and 10 µg/mL of insulin) for 2 days. The medium was replaced with DMEM containing 10% FBS and 10 µg/mL of insulin every other day. The cells were treated with or without PAT for 8 days during adipogenesis.

RAW 264.7 cells were obtained from the ATCC and maintained in high-glucose DMEM containing 10% FBS and 1% penicillin-streptomycin (10,000 U/mL). Cells were treated with 500 ng/mL of lipopolysaccharide (LPS, Sigma–Aldrich) in the presence or absence of PAT for 24 h to induce acute inflammatory response.

### 2.3. Cell Viability Assay

Cell viability was assessed using MTT assay. Briefly, 3T3-L1 cells were seeded in 96-well plates (2 × 10^4^ cells per well) containing DMEM supplemented with 10% BCS, followed by the addition of PAT to each well and culturing for 48 h. To evaluate the cytotoxicity of PAT on mature adipocytes, 3T3-L1 cells were allowed to differentiate into mature adipocytes, followed by treatment with various concentrations of PAT every 2 days. The medium was replaced with fresh medium containing insulin. MTT solution (0.5 mg/mL) was added to the incubation medium, followed by incubation for 4 h. After removing the medium, insoluble formazan crystals were dissolved in 100 μL of DMSO. The absorbance was measured at 570 nm using a microplate reader (Molecular Devices, Sunnyvale, CA, USA).

### 2.4. Oil Red O Staining

Lipid droplet accumulation in adipocytes was analyzed using Oil Red O (ORO, Sigma-Aldrich, St. Louis, MO, USA) staining. Briefly, cells were seeded in 24-well plates at a density of 5 × 10^4^ cells/well, and differentiation was induced. Differentiated cells were washed with Dulbecco’s phosphate buffered saline (DPBS, Welgene) twice and fixed with 3.7% formaldehyde solution (Biosesang, Yongin, Republic of Korea) for 15 min at 25 °C. After washing with 60% isopropanol (Ducksan, Ansan, Republic of Korea), the cells were stained with ORO solutions for 20 min at 25 °C. After removing the staining solution, the plate was washed with distilled water and dried, and cellular lipid droplets were imaged using an Olympus IX73 light microscope (Olympus, Center Valley, PA, USA). After microscopic observation, ORO staining was eluted with 100% isopropanol, and the absorbance was measured at 500 nm using a microplate reader (Molecular Devices).

### 2.5. Determination of TG Content

TG content was measured using a TG assay kit (#ab65336, Abcam, Cambridge, MA, USA), according to the manufacturer’s protocols. Briefly, cells were washed with DPBS and resuspended in 5% NP-40/ddH2O solution. TGs were solubilized by subjecting the samples to heating and cooling three times. Thereafter, lipase was introduced to convert TG into glycerol and fatty acid. The samples were centrifuged at 15,000 rpm for 3 min to collect soluble TG, and the absorbance was measured at 570 nm using a microplate reader (Molecular Devices). Individual values were normalized to the number of cells per well.

### 2.6. Measurement of Glycolipid Metabolism

Glucose concentration was measured using a glucose assay kit (#ab136955, Abcam). Briefly, the amount of glucose released into the culture medium by the cells was measured by adding the reaction mixture and incubating. Glucose concentration was determined by measuring the absorbance at 450 nm.

Glycerol released from mature 3T3-L1 cells into the medium was quantified using a glycerol assay kit (#MAK117, Sigma-aldrich, St. Louis, MI, USA). Briefly, the amount of glycerol released into the medium following the various treatments was measured by adding a reaction mixture, and the absorbance was measured at 570 nm.

LDL uptake was measured using an LDL uptake assay kit (#10011125, Cayman Chemicals, Ann Arbor, MI, USA). Briefly, cells were seeded in a black plate and stained with LDL-DyLight^TM^ solution in serum-free medium for 24 h. After removing the reaction medium, images were captured using a fluorescence microscope, and the fluorescence intensity was quantified using ImageJ (version 1.54d, NIH, Bethesda, MD, USA).

### 2.7. Protein Analysis

Cells were washed with DPBS and lysed with lysis buffer (Cell Signaling Technology) containing protease and phosphatase inhibitor cocktails (Roche, Basel, Switzerland). The lysate was centrifuged, and proteins were separated from lysate mixed with 5X SDS-PAGE buffer (Biosesang, Yongin, Republic of Korea) using SDS-PAGE and transferred to PVDF membrane (Bio-Rad, Hercules, CA, USA). After treatment with a blocking buffer (Bio-Rad, Hercules, CA, USA) to prevent nonspecific binding, the membranes were incubated with primary antibodies overnight at 4 °C, followed by washing with TBST and further incubation with secondary antibodies for 1 h at 25 °C. The proteins were detected using chemiluminescent substrate (Thermo Fisher Scientific, Sunnyvale, CA, USA), and densitometry was conducted using ImageJ (version 1.54d).

### 2.8. RNA Analysis

Total RNA was extracted from cell pellets using an RNeasy mini kit (Qiagen, Hilden, Germany), following the manufacturer’s instructions. Reverse transcription and removal of genomic DNA were conducted using a cDNA reverse transcription kit (TOYOBO CO., LTD., Osaka, Japan). Quantitative real-time polymerase chain reaction (qRT-PCR) was performed on a CFX ConnectTM Real-Time PCR detection system (Bio-Rad, Hercules, CA, USA) using equal amounts of cDNA mixed with Faststart Universal SYBR Green Master (Roche) and specific primers. The PCR conditions were as follows: 95 °C for 10 min, and 40 cycles at 95 °C for 20 s, 55 °C for 30 s, and 72 °C for 30 s. The primers used for RT-qPCR are listed in Table S1. The relative mRNA levels of target genes were normalized to those of the housekeeping genes (RPLP0, β-actin, GAPDH, or Eef2), and calculated using the ΔΔCt method.

### 2.9. Measurement of Mitochondrial Respiration

The real-time oxygen consumption rate (OCR) of the cells was measured using an extracellular flux analyzer (Seahorse XF, Agilent Technologies, Palo Alto, CA, USA). Briefly, cells were seeded in XF cell culture microplates and induced as described above. Thereafter, the culture medium was replaced with nonbuffered XF basal medium (#103575-100, Agilent Technologies, Santa Clara, CA, USA, XF DMEM, pH 7.4) supplemented with 25 mM of glucose, 1 mM of sodium pyruvate, and 4 mM of glutamine, after which the cells were allowed to degas in a non-CO_2_ incubator at 37 °C for 1 h prior to the measurement. Cellular OCR was determined before and after sequential injection of oligomycin (1.5 μM), carbonyl cyanide 4-trifluoromethoxy phenylhydrazone (FCCP; 1.0 μM), and antimycin A/rotenone (1.0 μM). The averaged OCR was monitored using the extracellular flux analyzer in a cycle consisting of mixing (150 s), waiting (120 s), and measuring (210 s). This cycle was repeated following each reagent injection. Mitochondrial respiration was determined by subtracting the nonmitochondrial OCR from the OCR after FCCP treatment. ATP production was determined by subtracting OCR after oligomycin treatment from the basal OCR. Finally, mitochondrial respiration data were normalized to those of the control group.

### 2.10. Mitochondrial Staining

Briefly, fixed cells were incubated with 200 nM of MitoTracker^TM^ Green FM (Invitrogen) or 500 nM of MitoTracker^TM^ Red CMXRos (Invitrogen, Waltham, MA, USA) in DPBS for 15 min at 37 °C. After washing with PBS, nuclei were stained with DAPI for 5 min, and fluorescence images were captured using a fluorescence microscope. The fluorescence intensity for mitochondrial mass and membrane potential were calculated using ImageJ (version 1.54d).

### 2.11. Pro-Inflammatory Cytokine and Nitric Oxide (NO) Quantification

The concentrations of pro-inflammatory cytokines (IL-6 and TNF-α) in RAW 264.7 cell culture medium were determined using BD OptEIA™ ELISA kits (BD biosciences, San Jose, CA, USA), following the manufacturer’s instructions. Briefly, specific capture antibodies for each cytokine were coated on 96-well immune plates (SPL Life sciences, Pocheon, Republic of Korea) and incubated overnight at 4 °C. After rinsing off excess antibodies, assay diluent was added to inhibit nonspecific binding, followed by the addition of each cytokine standard and cell culture supernatant at the designated concentrations. After incubation and washing, captured cytokines were further incubated with a mixture of biotin-conjugated detection antibodies and streptavidin-horse radish peroxidase conjugates (sAv-HRP). Thereafter, enzymatic reaction was initiated by adding the provided substrate solution, and the reaction was stopped by applying a stop solution (1 M H_3_PO_4_). Finally, the absorbance at 450 nm was measured using a microplate reader, and the standard curve was used to determine the cytokine concentration, expressed in ng/mL.

The concentration of NO produced in RAW 264.7 macrophages was quantified colorimetically using Promega Griess reagent (Madison, WI, USA), according to the manufacturer’s instructions. Briefly, each cell culture supernatant was mixed with 100 µL of mixed Griess reagent in a 96-well microplate and allowed to react for 10 min at 25 °C. NO concentration was determined by measuring the absorbance at 540 nm and comparing it to a standard curve established using sodium nitrite standards.

### 2.12. Intracellular ROS Quantification

Intracellular ROS levels were quantified using 2′,7′-dichlorofluorescin diacetate (DCFDA) flow cytometry. After treatment with LPS in the presence or absence of PAT for 24 h, RAW 264.7 cells were further incubated with 20 μM of DCFDA solution (#ab113851, Abcam) for 30 min at 37 °C in a 5% CO_2_ incubator. Thereafter, the cells were pelleted and washed with PBS two times, followed by the measurement of mean fluorescence intensity (MFI) using a CytoFLEX™ flow cytometer (Beckman Coulter, Brea, CA, USA).

### 2.13. Statistical Analysis

Data are presented as the mean ± standard error of the mean (SEM) of at least three replications. Statistical significance was determined using one-way analysis of variance (ANOVA), followed by Tukey’s post hoc test for multiple comparisons or unpaired two-tailed Student’s *t*-test in Prism 8 software (GraphPad Software, San Diego, CA, USA). Significant differences are indicated with different letters. Statistical significance was set at *p* < 0.05.

## 3. Results

### 3.1. PAT Alleviates Lipid Accumulation in Mature Adipocytes

**Figure 2:**
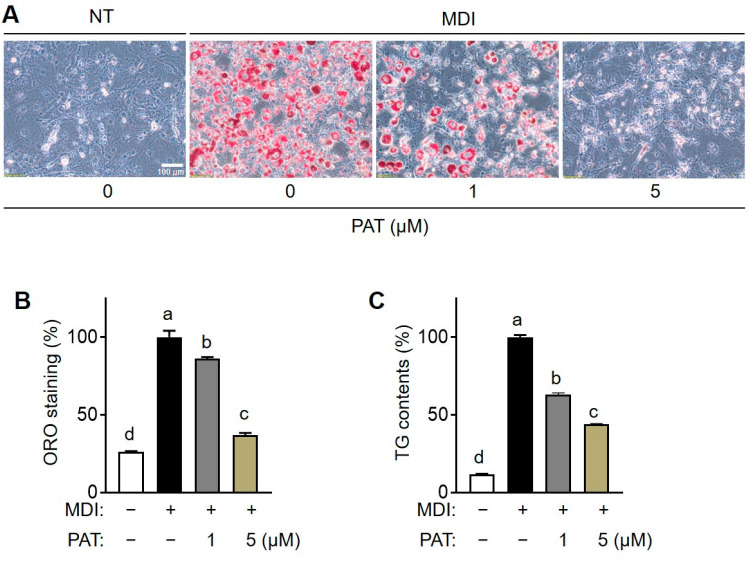
Effects of patulin (PAT) on lipid accumulation and triglyceride (TG) content in 3T3-L1 adipocytes. The 3T3-L1 cells were seeded and induced to differentiate in the presence of PAT (1 or 5 µM) for 8 days. (**A**) Cells were stained with Oil Red O to observe lipid droplets. (**B**) Oil Red O-stained cells were extracted using isopropanol, and lipid content was quantified at 550 nm using a spectrophotometer. (**C**) Total TG content was measured using a TG assay kit. Data are presented as the mean ± standard deviation (SD; *n* ≥ 3). Different letters indicate significantly different values at *p* < 0.05, as determined by one-way ANOVA followed by Tukey’s post hoc test.

Cell viability assay was performed to elucidate the cytotoxicity of different concentrations of PAT (0.2–10 µM) on preadipocytes and mature adipocytes. Compared with that in the untreated group, PAT was not cytotoxic to preadipocytes and matured adipocytes at concentrations ≤10 µM (Figure 1B). Therefore, we investigated the effects of 1 or 5 μM of PAT on lipid accumulation during adipocyte differentiation. Adipocyte maturation is governed by insulin signaling, which promotes the uptake of glucose and free fatty acids (FFAs), leading to the accumulation of TGs [28]. Thus, we investigated the effects of PAT on lipid deposition in adipocytes using ORO staining (Figure 2A). ORO staining intensity was notably higher in the differentiated group than in the undifferentiated group, indicating enhanced lipid accumulation during differentiation. Notably, PAT treatment caused a dose-dependent decrease in ORO staining intensity (Figure 2B). Additionally, PAT treatment induced a dose-dependent decrease in elevated levels of TG in differentiated 3T3-L1 cells compared with that in the control group (Figure 2C). These findings suggest that PAT effectively inhibits lipid accumulation during the differentiation of 3T3-L1 preadipocytes without adversely affecting their viability.

### 3.2. PAT Modulates Glycolipid Metabolism in Adipocytes

**Figure 3:**
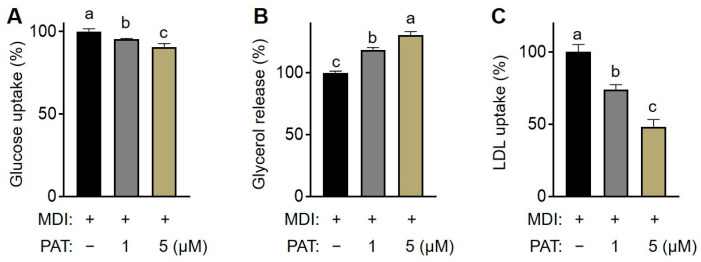
Effect of patulin (PAT) on glycolipid metabolism. The 3T3-L1 cells were cultured and induced to differentiate in the presence of PAT (1 or 5 µM) for 8 days. (**A**,**B**) The culture medium was collected, and the amount of glucose and glycerol in the medium was measured. (**C**) LDL uptake was examined under a fluorescence microscope. The relative fluorescence intensity per cell was quantified using ImageJ (version 1.54d). Data are presented as the mean ± standard deviation (SD; *n* = 3). Different letters indicate significantly different values at *p* < 0.05, as determined using one-way ANOVA followed by Tukey’s post hoc test.

To elucidate the mechanism of PAT in lipid accumulation in mature 3T3-L1 cells, we investigated changes in glucose uptake, glycerol release, and LDL uptake following PAT treatment. Specifically, the levels of glucose and glycerol in the cell culture medium were determined after treatment with PAT (1 or 5 µM). PAT treatment significantly suppressed glucose uptake in a dose-dependent manner, as evidenced by a higher glucose content in the PAT treatment group than in the untreated group (Figure 3A). Additionally, PAT treatment significantly increased glycerol concentration in the culture medium (Figure 3B) and induced a dose-dependent decrease in LDL uptake compared with that in the control group (Figure 3C). Overall, these results indicate that PAT regulates glycolipid metabolism by inhibiting glucose and LDL uptake and promoting the release of glycerol in mature adipocytes.

### 3.3. PAT Suppresses the Expression of Adipogenic/Lipogenic Genes

**Figure 4:**
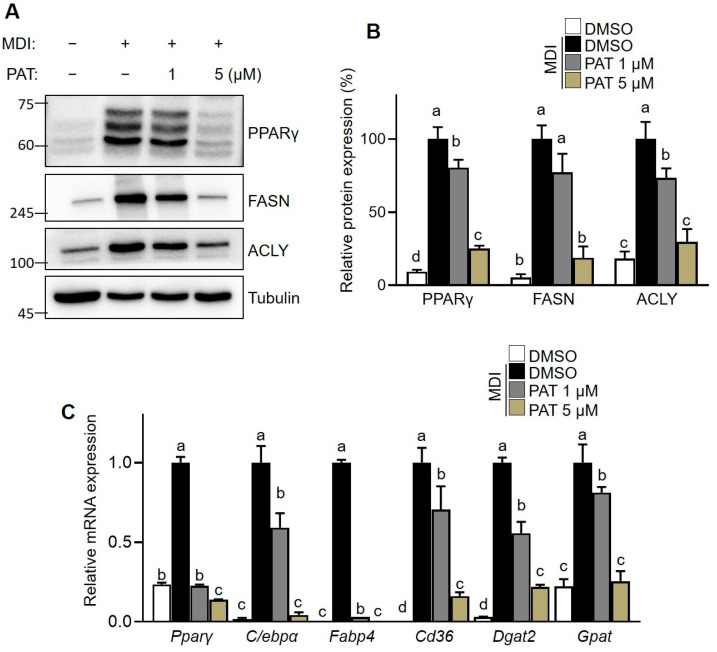
Effects of patulin (PAT) on the expression of lipid-metabolism-related proteins and key metabolic regulators in 3T3-L1 adipocytes. The 3T3-L1 cells were seeded and induced to differentiate in the presence of PAT (1 or 5 µM) for 8 days. (**A**) Protein expression levels of PPARγ, FASN, and ACLY were analyzed using immunoblotting assay. (**B**) Bar graphs indicate the densitometric quantification of PPARγ/α-tubulin, FASN/α-tubulin, and ACLY/α-tubulin bands. Data are expressed as the mean ± standard deviation (SD; *n* = 3). (**C**) mRNA expression of genes related to adipogenesis/lipogenesis in 3T3-L1 adipocytes were analyzed using RT-qPCR. Data are expressed as the mean ± SD (*n* = 3). Different letters within each column indicate significant differences at *p* < 0.05 levels between treatments, as determined by one-way ANOVA followed by Tukey’s post hoc test (**B**,**C**).

Furthermore, we investigated the effect of PAT on the expression of adipogenic and lipogenic mediators in 3T3-L1 adipocytes. There was a significant increase in PPARγ, FASN, and ACLY protein expression levels following the differentiation of 3T3-L1 preadipocytes into mature adipocytes (Figure 4A). However, PAT treatment significantly decreased the expression of the proteins in a dose-dependent manner (Figure 4B). Additionally, RT-qPCR showed that PAT treatment significantly suppressed the upregulation of key adipogenic/lipogenic genes, including *Pparγ*, *C/ebpα*, *Fabp4*, *Cd36*, *Dgat2*, and *Gpat*, during the differentiation process compared with that in the control group (Figure 4C). These results indicate that PAT exerts an inhibitory effect on the expression of adipogenic and lipogenic genes.

### 3.4. PAT Increases the Expression of Fatty Acid Oxidation-Related Genes

**Figure 5:**
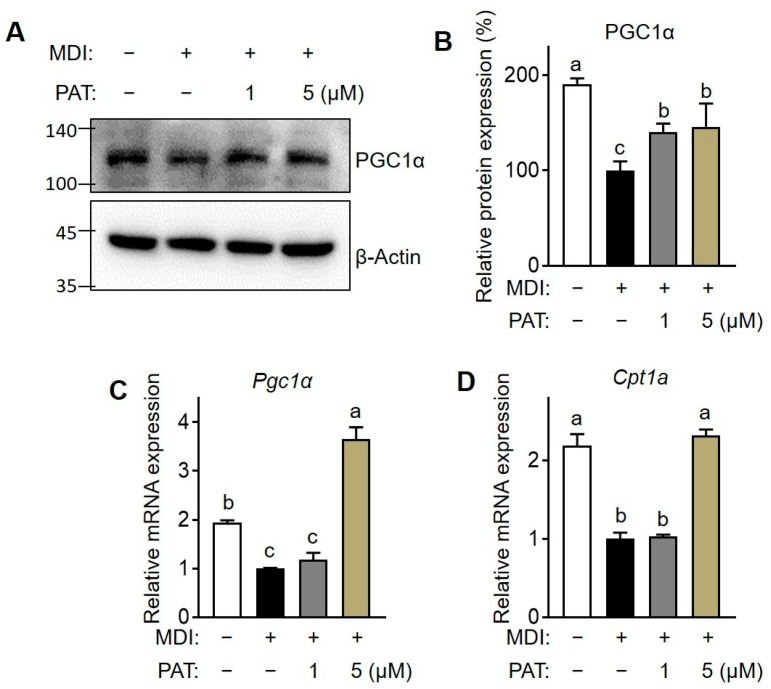
Effects of patulin (PAT) on the expression of fatty acid oxidation-related genes in 3T3-L1 adipocytes. The 3T3-L1 cells were cultured and induced to differentiate in the presence of PAT (1 or 5 µM) for 8 days. (**A**) Protein expression levels of PGC1α were determined by immunoblotting. (**B**) The bar graph indicates the relative intensity of PGC1α/ β-Actin bands. mRNA expression levels of (**C**) Pgc1α and (**D**) Cpt1a were analyzed using RT-qPCR. Data are expressed as the mean ± standard deviation (SD; *n* = 3). Different letters indicate significantly different values at *p* < 0.05, as determined by one-way ANOVA followed by Tukey’s post hoc test.

To evaluate the effect of PAT on fatty acid oxidation (FAO), we examined the expression of PGC1α and related molecules at the protein and mRNA levels. Compared with that in undifferentiated 3T3-L1 adipocytes, there was a decrease in PGC1α mRNA and protein expression in matured adipocytes. However, treatment with 5 µM of PAT stimulated PGC1α expression (Figure 5A–C) and significantly increased the expression of *Cpt1a* gene compared with that in untreated control group (Figure 5D). Overall, these results suggest that PAT inhibits adipogenesis by promoting FAO.

### 3.5. PAT Enhances Mitochondrial Function in Adipocytes

**Figure 6:**
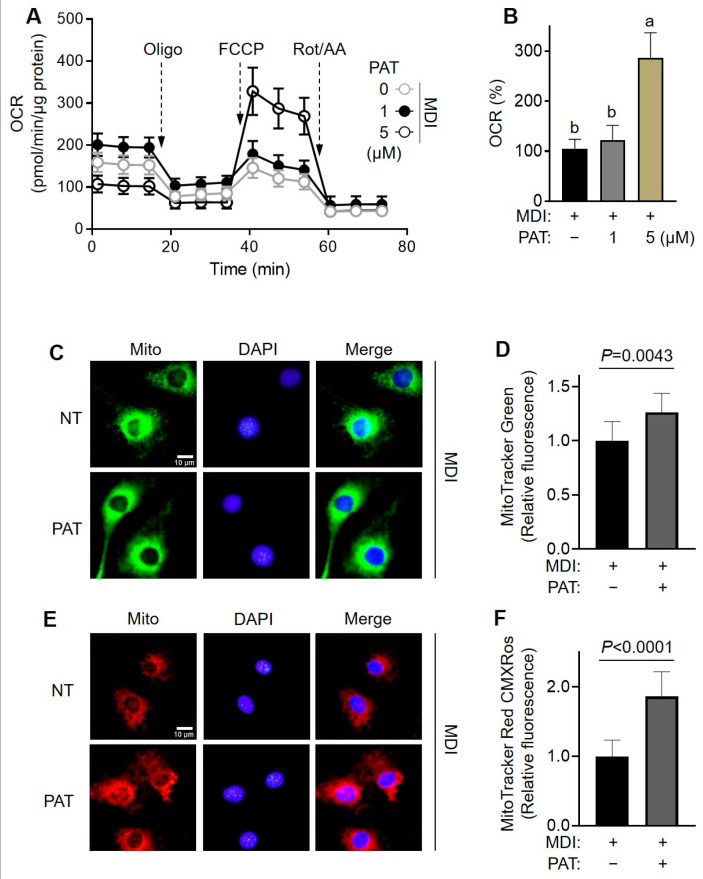
Effects of patulin (PAT) on mitochondrial oxidative function in 3T3-L1 adipocytes. The 3T3-L1 cells were seeded and induced to differentiate in the presence of PAT (1 or 5 µM) for 8 days. (**A**,**B**) Oxygen consumption rate (OCR) was measured as described in the Materials and Methods section. Representative data from three independent experiments are presented. The indicated values were normalized to the protein content determined using BCA assay. (**B**) Different letters indicate significantly different values at *p* < 0.05, as determined using one-way ANOVA followed by Tukey’s post hoc test. (**C**,**E**) Representative images of MitoTracker Green-stained adipocytes (**C**, for mitochondrial mass) and CMXRos-stained adipocytes (**E**, for mitochondrial membrane potential, ΔΨm) are shown. (**D**,**F**) Quantitative analysis of fluorescence intensity is shown. Statistical significance (*p* value) was determined using unpaired two-tailed Student’s *t*-test.

PGC1α plays a pivotal role in mitochondrial metabolism, promoting mitochondrial biogenesis and bioenergetics [29]. Therefore, we investigated the effects of PAT treatment on mitochondrial respiration in mature 3T3-L1 adipocytes by measuring OCR using Seahorse analysis. Notably, treatment with 5 µM of PAT significantly increased cellular OCR compared with that in the untreated group (Figure 6A,B). To further elucidate the impact of PAT on mitochondrial integrity, we examined changes in mitochondrial mass and membrane potential using MitoTracker^TM^ Green and Red staining, respectively (Figure 6C–F). PAT-treated cells exhibited more intense green staining compared with untreated cells, indicative of an increase in mitochondrial mass in mature adipocytes (Figure 6C,D). Additionally, PAT treatment increased mitochondrial membrane potential in mature adipocytes. Moreover, flow cytometry confirmed that PAT treatment not only elevated mitochondrial mass but also improved mitochondrial membrane integrity (Figure S1). Collectively, these results indicate that PAT positively influences mitochondrial functions by improving both mitochondria mass and quality in mature adipocytes.

### 3.6. PAT Inhibits the Secretion of Pro-Inflammatory Cytokines in Macrophages

**Figure 7:**
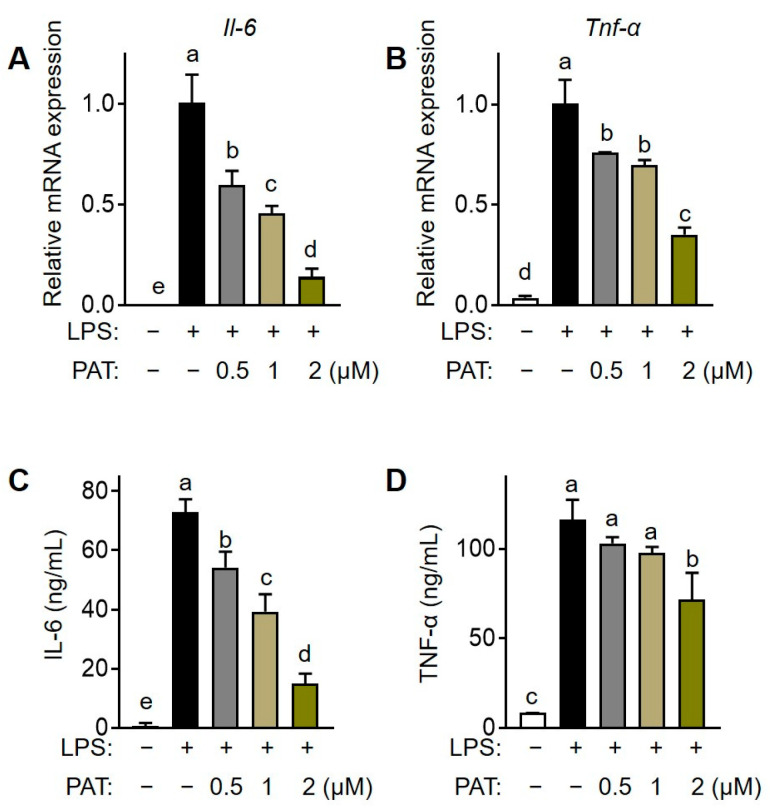
Effects of patulin (PAT) on pro-inflammatory cytokine production in lipopolysaccharide (LPS)-induced macrophages. RAW 264.7 cells were preincubated and further exposed to LPS (500 ng/mL) in the presence of PAT (1 or 5 µM) for 24 h. (**A**,**B**) The transcription of pro-inflammatory genes (*Il-6* and *Tnf-α*) in RAW 264.7 cells was assessed using RT-qPCR. (**C**,**D**) The production of pro-inflammatory cytokines (IL-6 and TNF-α) in cell supernatants was quantified using ELISA. Different letters indicate significantly different values at *p* < 0.05, as determined using one-way ANOVA followed by Tukey’s post hoc test.

Macrophages are crucial immune cells involved in obesity-associated chronic low-grade inflammation [30]. In addition to its antilipidemic effect in 3T3-L1 adipocytes, we investigated the effect of PAT in inflammatory response in LPS-treated RAW 264.7 macrophages. MTT assay demonstrated that PAT was not cytotoxic to RAW 264.7 cells at concentrations ≤2 µM (Figure S2). Additionally, PAT treatment significantly suppressed LPS-induced transcription of the pro-inflammatory *Tnf-α* and *Il-6* genes in a dose-dependent manner (Figure 7A,B). Moreover, the LPS-induced increase in the secretion of both IL-6 and TNF-α was significantly inhibited by PAT treatment in a dose-dependent manner compared with that in the untreated group (Figure 7C,D). Overall, these results indicate that PAT reduces pro-inflammatory cytokine production in LPS-induced macrophages.

### 3.7. PAT Reduces Inflammatory Mediator Production in Macrophages

**Figure 8:**
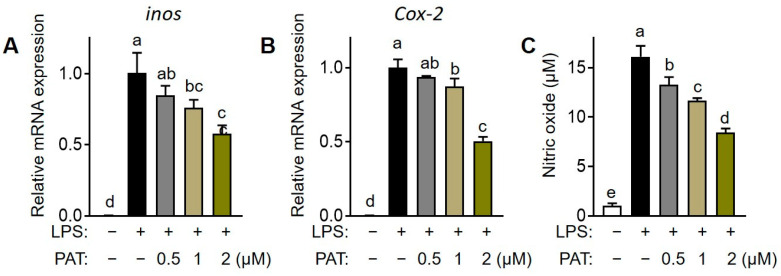
Effects of patulin (PAT) on inflammatory gene transcription and inflammatory mediator production in lipopolysaccharide (LPS)-induced macrophages. (**A**,**B**) mRNA expression of *inos* and *Cox-2* in RAW 264.7 cells were analyzed using RT-qPCR. (**C**) The production of nitric oxide (NO) was quantified by colorimetric assay using Griess reagent. Different letters indicate significantly different values at *p* < 0.05, as determined using one-way ANOVA followed by Tukey’s post hoc test.

Furthermore, we investigated the effects of PAT on inflammatory mediators, including inducible nitric oxide synthase (iNOS) and cyclooxygenase-2 (COX-2). PAT treatment at concentrations ≥1 µM significantly inhibited LPS-induced upregulation in the mRNA expression of *inos* and *Cox-2* (Figure 8A,B). Moreover, PAT treatment significantly suppressed the LPS-induced increase in the production of nitric oxide (NO), a byproduct of inos, in a dose-dependent manner compared with that in the untreated group (Figure 8C). These findings suggest that PAT may improve inflammatory response by inhibiting the production of pro-inflammatory cytokines and mediators in LPS-stimulated macrophages.

### 3.8. PAT Modulates Mitochondrial Function in Macrophage

**Figure 9:**
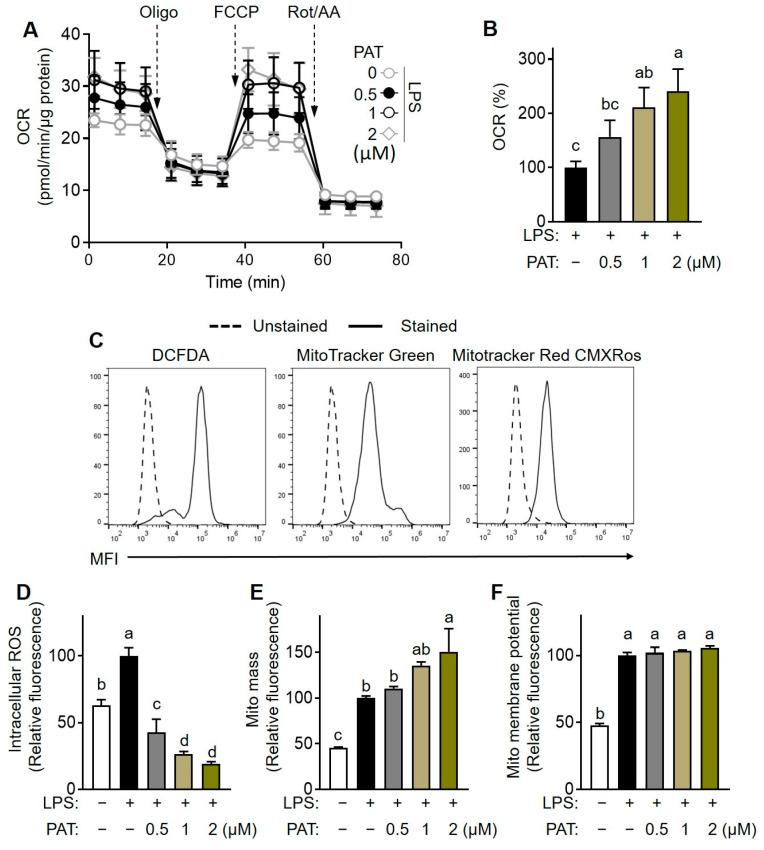
Effects of patulin (PAT) on mitochondrial oxidative function in lipopolysaccharide (LPS)-induced RAW 264.7 macrophages. (**A**,**B**) Oxygen consumption rate (OCR) was measured as described in the Materials and Methods section. Representative data from three independent experiments are presented. The indicated values were normalized to the protein content, determined using BCA assay. (**C**) Flow cytometry was performed to examine macrophages stained with DCFDA, MitoTracker Green, and MitoTracker Red CMXRos. (**D**) Intracellular ROS levels, (**E**) mitochondria (Mito) mass, and (**F**) Mito membrane potential were determined using mean fluorescence intensity (MFI) obtained through flow cytometry, employing specific probes for staining. The data were normalized to the values of LPS-treated control. Different letters indicate significantly different values at *p* < 0.05, as determined using one-way ANOVA followed by Tukey’s post hoc test.

The mitochondrial energy metabolic phenotype of macrophages plays a crucial role in shaping their function and immunophenotype [31]. Thus, we investigated the effects of PAT on mitochondrial respiration in macrophages by measuring the OCR. LPS stimulation decreased mitochondrial respiration and ATP production in macrophages considerably (Figure S3). Notably, PAT treatment at concentrations ≥1.0 μM enhanced both mitochondrial respiration (Figure 9A,B) and ATP production (Figure S4). Furthermore, flow cytometry analysis was performed to assess intracellular ROS production, mitochondrial mass, and mitochondrial membrane potential (Figure 9C–F). PAT treatment significantly reversed LPS-induced increase in ROS production in a dose-dependent manner (Figure 9D) and significantly increased mitochondrial mass compared with that in the untreated LPS-stimulated group (Figure 9E). However, PAT treatment did not significantly affect mitochondrial membrane potential in LPS-stimulated macrophages (Figure 9F). Collectively, these results suggest that the enhancement of mitochondrial bioenergetics contributes to the anti-inflammatory effect of PAT, likely mediated by its antioxidant properties.

## 4. Discussion

Research evidence suggests that chronic obesity is accompanied by the accumulation of macrophages, contributing to the development of insulin resistance in adipose tissue, skeletal muscle, and the liver [32]. Despite this understanding, the exact cause of inflammation in obesity remains poorly understood, and effective targeted therapeutic agents are limited. Currently, lifestyle improvements, such as changes in diet and exercise, are commonly adopted strategies to combat obesity. However, achieving and maintaining these behavioral changes can be challenging, making drug options a consideration [33]. Although various antiobesity drugs are currently available, their long-term use can lead to serious side effects [34]. Thus, studies are necessary to establish a link between the consumption of safe natural food ingredients and a decrease in obesity and related complications [35]. Research evidence indicates that the consumption of natural products is associated with reduced risk of obesity-related complications [36]. Given the increasing recognition that safe alternatives derived from natural sources may be effective in alleviating obesity, extensive research is necessary to identify natural compounds with antiobesity effects. Therefore, this study elucidated the antilipidemic and anti-inflammatory effects of PAT, a natural compound derived from fungi, through in vitro experiments.

Adipose tissues are insulin-sensitive peripheral tissues, secreting various hormones to maintain overall energy homeostasis in the body [37]. The regulation of adipogenesis entails an intricate transcriptional cascade that includes the sequential activation of C/EBP and PPARγ at the molecular level [38]. In adipocytes, PPARγ and C/EBPα act synergistically to promote the synthesis and transport of FFAs by sharing binding sites [38]. This synergistic action is essential for lipid metabolism and proper functioning of adipose tissue in maintaining energy balance [39]. Additionally, both PPARγ and C/EBPα are essential for driving insulin-stimulated glucose uptake and can induce excessive production of FFAs in obese individuals [40]. Therefore, regulating transcriptional networks in differentiating adipocytes could be a promising therapeutic strategy for FFA-induced insulin resistance and obesity. In the present study, PAT treatment decreased the protein and mRNA expression of PPARγ and C/EBPα in differentiated 3T3-L1 cells, a commonly used model for studying adipocyte differentiation. Additionally, PAT treatment suppressed insulin-induced upregulated of lipogenic genes, glucose and LDL uptake, lipid droplet accumulation, and TG levels, suggesting that PAT can influence adipogenesis and lipogenesis by modulating the expression of key transcription factors involved in insulin-induced adipocyte differentiation. However, further studies are necessary to comprehensively understand the specific molecular pathways through which PAT inhibits lipid accumulation in mature adipocytes.

During periods of increased energy demand, TGs stored in adipose tissues are broken down into FFAs and glycerol, which serve as energy for other organs [41]. This process involves the proportional release of glycerol and FFAs from adipocytes, making glycerol release from mature adipocytes an important index for assessing lipolysis [41]. FFAs are further oxidized to provide energy through mitochondrial FAO, and the PGC1-α plays a crucial role in activating mitochondrial biogenesis and FAO [42]. Additionally, the transcriptional activity of PGC-1α is responsible for regulating the expression of gene networks that control glucose consumption, the tricarboxylic acid cycle, and oxidative phosphorylation (OXPHOS) in adipose tissue [43]. Considering that mitochondrial dysfunction in adipocytes is closely associated with insulin resistance in obese individuals [44], enhancing mitochondrial function through the activation of PGC1-α could be a promising strategy to prevent obesity and related metabolic complications.

Several studies have demonstrated that natural compounds can exert protective effects against obesity by activating PGC1-α. For instance, zeaxanthin, a carotenoid found in various vegetables, enhanced PGC1-α expression and mitochondrial respiration, leading to decreased lipid accumulation in 3T3-L1 adipocytes [45]. Additionally, ginsenoside Rb1 increased PGC1-α expression and mitochondrial oxidative function, including ATP generation, in mature adipocytes, thereby improving glycolipid metabolism and reducing weight gain in obese mice [46]. Moreover, sesamol, the main phenolic compound derivative in sesame oil, suppressed high-fructose-diet-induced lipogenesis and bodyweight gain by upregulating mitochondrial metabolism-related genes, including PGC1-α, in mature adipocytes [47]. In the present study, treatment with 5 μM of PAT significantly elevated glycerol secretion and increased the protein and mRNA expression of PGC1-α and its FAO downstream target gene *Cpt1a* in differentiated 3T3-L1 adipocytes, indicating an induction of the breakdown of FFAs. Moreover, PAT treatment had a notable impact on mitochondrial function by significantly enhancing mitochondrial oxidative capacity, increasing mitochondrial mass, and improving mitochondrial membrane integrity. Overall, these findings suggest that PAT has the potential to improve mitochondrial function. Based on its positive effects on mitochondrial integrity, PAT could serve as a promising therapeutic for ameliorating obesity and its associated metabolic dysregulations.

Adipose tissue, a substantial endocrine organ harboring several immune cells, particularly macrophages, plays a crucial role in both health and disease conditions by clearing apoptotic adipocytes and facilitating tissue remodeling to maintain homeostasis [48]. In the obese state, the population of macrophages in adipose tissue increases by more than three times compared with that in the lean state, and these macrophages interact with adipocytes, leading to an inflammatory phenotype in obese individuals [49]. During this process, adipocytes detect danger signals mimicking microbial infections and trigger inflammatory responses by activating the toll-like receptor (TLR) signaling pathway, resulting in the release of pro-inflammatory mediators, such as NO and cytokines [50]. Notably, the TNF-α level was upregulated in the adipose tissue of obese subjects, and the inhibition of TNF-α expression was closely associated with weight loss in these individuals [9]. Therefore, downregulating inflammatory cytokines and mediators produced by activated macrophages could be a beneficial approach for preventing obesity-related chronic inflammation. In the present study, RAW 264.7 cells were stimulated with LPS to induce oxidative stress and inflammatory signaling cascade through TLRs on macrophages [51], and the anti-inflammatory effect of PAT was evaluated. Consistent with our data, PAT demonstrated the potential to suppress NO production by downregulating mitogen-activated protein kinase (MAPK) signaling pathway in LPS-induced murine peritoneal macrophages [26]. Additionally, PAT treatment inhibited IL-6 and TNF-α expression, as well as the expression of inducible enzyme-encoding genes, such as *Cox-2*, under conditions of acute inflammation.

Recent research findings suggest a close relationship between mitochondrial functions and the development and progression of metabolic diseases, including obesity [52]. Moreover, the intricate interplay of metabolic and immune responses, regulated by macrophage mitochondria, has been emphasized due to the crucial role of these responses in assessing the inflammatory states [31]. In physiological conditions, healthy mitochondria maintain a balance between the formation and elimination of ROS through their antioxidant defense systems [53]. However, prolonged inflammatory conditions can alter the equilibrium between antioxidants and free radicals, leading to abnormal ROS generation, which triggers an increase in the production of pro-inflammatory mediators and contributes to oxidative stress and development of the chronic state [54]. Moreover, overnutrition is associated with chronic oxidative and inflammatory stress in individuals with obesity [55], suggesting that inhibiting excessive ROS production by maintaining mitochondrial oxidative function could be a promising therapeutic approach to alleviate obesity-related inflammatory diseases. In this study, PAT treatment increased mitochondrial respiration, ATP production, and mitochondrial mass, and it suppressed intracellular ROS generation in LPS-stimulated macrophages. Typically, mitochondrial mass and ROS generation exhibit a proportional relationship. However, the discrepancy in our results might be attributed to PAT-induced improvement in antioxidant capacity via upregulation of mitochondrial biogenesis. This notion is supported by the upregulation of the PGC1α gene expression in PAT-treated 3T3-L1 adipocytes. Consistent with our findings, a previous study demonstrated that PGC1α enhanced both mitochondrial mass and the expression of primary mitochondrial antioxidant enzymes, such as SOD2 [56]. Overall, these findings suggest that PAT exerts antioxidant and anti-inflammatory effects by enhancing mitochondrial oxidative capacity, making it a promising candidate for mitigating chronic diseases, including obesity.

Although our study demonstrated the anti-inflammatory efficacy of PAT in LPS-induced macrophages by upregulating mitochondrial function, its direct effect on macrophages in adipose tissue remains unclear. Thus, further research is necessary to investigate the effects of PAT on obesity-related inflammation using an in vitro coculture system. Additionally, in vivo obesity models are necessary to explore the effects of PAT on macrophage polarization and its bidirectional interplay with adipocytes. This approach would enable the investigation of the crosstalk between macrophages and adipocytes through a paracrine loop, shedding light on the potential impact of PAT in obesity.

The cytotoxic and immune-toxic impacts of PAT remain topics of debate. PAT has been noted to decrease cell viability in both murine RAW 264.7 macrophages and human erythrocytes at concentrations > 5 μM [57,58]. However, PAT did not induce any cytotoxicity at concentrations ranging from 1 to 5 μM in the present study. Notably, we demonstrated the antilipidemic and anti-inflammatory effects of PAT using hypertrophied 3T3-L1 adipocytes and RAW 264.7 macrophages, respectively. Moreover, treatment with ≤2.56 mg/kg of PAT did not cause any adverse effects on parameters, such as body weight and the weights of organs including the liver, spleen, and thymus, in mice [59]. Consequently, there is a compelling need for additional animal studies to explore the potential benefits of utilizing PAT, with careful consideration of its possible toxicological implications.

Conclusively, these findings indicate that PAT exerts antiobesity effects by inhibiting lipid accumulation and promoting fatty acid oxidation in hypertrophied adipocytes. Additionally, PAT effectively mitigates inflammation by regulating pro-inflammatory mediators, cytokines, and ROS levels. These beneficial effects are closely associated with the modulation of mitochondrial integrity and its oxidative functions. Therefore, PAT could serve as a potent modulator for preventing or ameliorating obesity-related chronic inflammatory diseases. However, animal models and clinical studies are necessary to validate the antiobesity effects of PAT.

## Data Availability

The data presented in this study are available upon request from the corresponding author.

## Supplementary Materials

The following supporting information can be downloaded at: https://www.mdpi.com/article/10.3390/antiox12091750/s1, Figure S1: Analysis of mitochondrial functions in 3T3-L1 adipocytes; Figure S2: Effects of patulin (PAT) on RAW 264.7 cell viability; Figure S3: Effects of lipopolysaccharide (LPS) treatment on the mitochondrial oxidative function in RAW 264.7 macrophages; Figure S4: Effects of patulin (PAT) on the mitochondrial ATP production in lipopolysaccharide (LPS)-induced RAW 264.7 macrophages; Table S1: Primer sequences of mouse mRNA used in RT-PCR analysis.
