# Supplementary material for: Patulin Ameliorates Hypertrophied Lipid Accumulation and Lipopolysaccharide-Induced Inflammatory Response by Modulating Mitochondrial Respiration
Source: Antioxidants (Basel). 2023 Sep 11;12(9):1750. doi: 10.3390/antiox12091750 (PMC10526082; doi:10.3390/antiox12091750)
Supplement: Supplementary file 1 [file antioxidants-12-01750-s001.zip › antioxidants-2589156-Supplymentary.pdf]

antioxidants

Supplementary Materials For

Patulin Ameliorates Hypertrophied Lipid Accumulation and Lipopolysaccharide-Induced Inflammatory Response by Modulating Mitochondrial Respiration

Seulmin Hong [1,2], Seon Kyeong Park [1], Jangho Lee [1], Soo Hyun Park [1], Young-Soo Kim [2], Jae-Ho Park [1], Seungmin Yu [1,*] and Yu Geon Lee [1,*]

[1] Personalized Diet Research Group, Korea Food Research Institute (KFRI), Wanju 55365, Republic of Korea
[2] Department of Food Science & Technology, Chonbuk National University, Jeonju 54896, Republic of Korea
* Correspondence: y.seungmin@kfri.re.kr (S.Y); ugun2@kfri.re.kr (Y.G.L.); Tel.: +82-63-219-9565 (S.Y); +82-63-219-9585 (Y.G.L.)

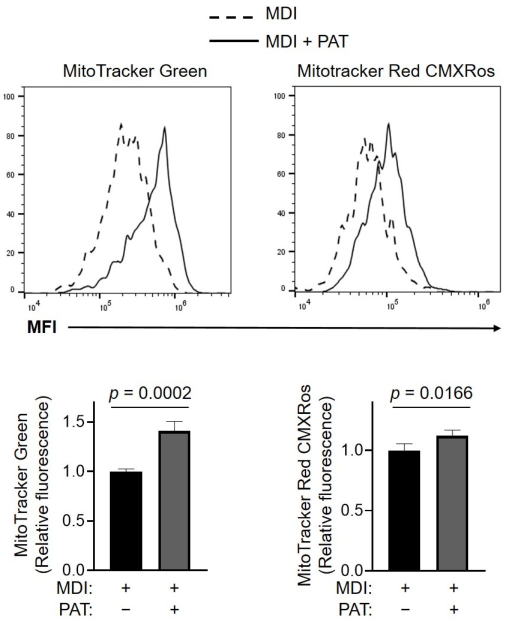

Figure S1. Analysis of mitochondrial function in 3T3-L1 adipocytes. 3T3-L1 cells were seeded and induced to differentiate in the presence of patulin (PAT, 1 or 5 μM) for 8 days. Mitotracker Green and Mitotracker red-stained cells were detected using flow cytometer, and the relative fluorescence intensity was calculated by normalizing to the MFI values of the PAT-untreated control. Statistical significance (*p* value) was determined using unpaired two-tailed Student's *t*-test.

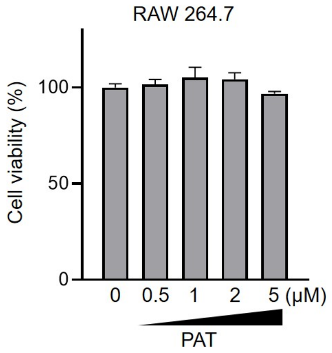

Figure S2. Effects of patulin (PAT) on RAW 264.7 cell viability. RAW 264.7 cell were incubated in a medium containing different concentrations of PAT for 6 h. Cell viability was measured using WST-1 kit.

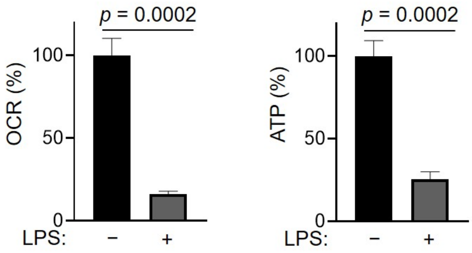

Figure S3. Effects of lipopolysaccharide (LPS) treatment on mitochondrial oxidative function in RAW 264.7 macrophages. Oxygen consumption rate (OCR) and ATP production levels were determined as described in the Materials and Methods. The data were normalized to the OCR values of the unstimulated control. Statistical significance (*p* value) was determined using unpaired two-tailed Student's *t*-test.

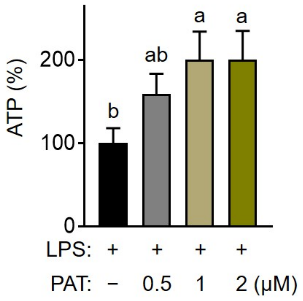

Figure S4. Effects of patulin (PAT) on the mitochondrial ATP production in lipopolysaccharide (LPS)-induced RAW 264.7 macrophages. ATP generation was calculated as described in the Materials and Methods. The data were normalized to calculated OCR values for the PAT-untreated control. Different letters indicate significantly different values at $p < 0.05$, as determined by one-way ANOVA followed by Tukey's post-hoc test.

Table S1. Primer sequences of mouse mRNA used in RT-PCR analysis

Gene	Direction	Sequence (5'-3')
Pparγ	forward<br>reverse	GCGGGCTGAGAAGTCACGTT<br>CCATCACGGAGAGGTCCACA
C/ebpα	forward<br>reverse	TACCGAGTAGGGGGAGCA<br>TCATTTTTCTCACGGGGCC
Fasn	forward<br>reverse	AGAAGCCATGTGGGGAAGATT<br>AGCAGGGACAGGACAAGACAA
Acly	forward<br>reverse	CTTGGGCCGGAACAAAA<br>GCCGAGGTGGTGCAGAT
Fabp4	forward<br>reverse	GGGAACCTGGAAGCTTGTCT<br>ACTCTCTGACCGGATGGTGA
Cd36	forward<br>reverse	GTGCAAAACCCAGATGACGT<br>TCCAACAGACAGTGAAGGCT
Dgat1	forward<br>reverse	GGCCCAAGGTAGAAGAGGAC<br>GATCAGCATCACCACACACC
Gpat	forward<br>reverse	GTAGTTGAACTCCTCCGACA<br>ATCCACTACCACTGAGAGGA
Pgc1α	forward<br>reverse	AAGTGGTGTAGCGACCAATCG<br>AATGAGGGCAATCCGTCTTCA
Cpt1a	forward<br>reverse	CTCCGCCTGAGCCATGAAG<br>CACCAGTGATGCCATTCT
Il-6	forward<br>reverse	CAAAGCCAGAGTCCTTCAGA<br>TTGGTCCTTAGCCACTCCTT
Tnf-α	forward<br>reverse	AGCCCACGTCGTAGCAAACCAC<br>AGGTACAACCCATCGGCTGGCA
inos	forward<br>reverse	CCTGTGTTCCACCAGGAGAT<br>CCCTGGCTAGTGCTTCAGAC
Cox-2	forward<br>reverse	TGACCCCCAAGGCTCAAAT<br>GAACCCAGGTCCTCGCTTATG
Rplp0	forward<br>reverse	AGGTCCTCCTTGGTGAAC<br>GTGCTGATGGGCAAGAAC
Gapdh	forward<br>reverse	AGTATGACTCCACTCACGGCAAAT<br>GTCTCGCTCCTGGAAGATGGT
β-actin	forward<br>reverse	GGCTGTATTCCCCTCCATCG<br>CCAGTTGGTAACAATGCCATGT
Eef2	forward<br>reverse	CGGGACACGGCTCTTAACAT<br>CTTCCTGGAGGCACTTACCC

Note: peroxisome proliferator-activated receptor-γ, Ppar-γ; CCAAT/enhancer-binding protein α, C/ebpα; fatty acid synthase, Fasn; ATP-citrate lyase, Acly; fatty acid-binding protein 4, Fabp4; cluster of differentiation 36, Cd36; diacylglycerol O-acyltransferase 1, Dgat1; glycerol-3-phosphate O-acyltransferase, Gpat; Peroxisome proliferator-activated receptor γ coactivator 1 α, Pgc1α; carnitine palmitoyltransferase 1α, Cpt1α ; interleukin-6, Il-6; tumor necrosis factor-α, Tnf-α; inducible nitric oxide synthase, inos; cyclooxygenase-2, Cox-2.
